# Comparison of Electronic Surveillance With Routine Monitoring for Patients With Lymphoma at High Risk of Relapse: Prospective Randomized Controlled Phase 3 Trial (Sentinel Lymphoma)

**DOI:** 10.2196/65960

**Published:** 2025-05-06

**Authors:** Katell Le Dû, Adrien Chauchet, Sophie Sadot-Lebouvier, Olivier Fitoussi, Bijou Fontanet, Arnaud Saint-Lezer, Frédéric Maloisel, Cédric Rossi, Sylvain Carras, Anne Parcelier, Magali Balavoine, Anne-Lise Septans

**Affiliations:** 1 Department of Hematology, Confluent Private Hospital, 2 rue Eric Tabarly, Nantes, 44277, France, 33 0615246067; 2 Department of Hematology, University Hospital, Besançon, France; 3 Department of Hematology, Polyclinique Bordeaux Nord Aquitaine, Bordeaux, France; 4 Department of Hematology, Bergonié Institute, Bordeaux, France; 5 Department of Hematology, Mont de Marsan Hospital, Mont de Marsan, France; 6 Department of Hematology, Clinique Saint-Anne, Strasbourg, France; 7 Department of Hematology, University Hospital, Dijon, France; 8 Department of Hematology, University Hospital, Grenoble, France; 9 Department of Hematology, Centre hospitalier Bretagne Atlantique, Vannes, France; 10 Department of Biostatistics, Institut inter-régional de cancérologie, Le Mans, France

**Keywords:** patient-reported outcome measures, lymphoma, risk of relapse, relapse, randomized trial, web-based, quality of life, survival, detection, progression, T-cell lymphoma, Hodgkin lymphoma

## Abstract

**Background:**

Relapse is a major event in patients with lymphoma. Therefore, early detection may have an impact on quality of life and overall survival. Patient-reported outcome measures have demonstrated clinical benefits for patients with lung cancer; however, evidence is lacking in patients with lymphoma. We evaluated the effect of a web-mediated follow-up application for patients with lymphoma at high risk of relapse.

**Objective:**

This study aims to demonstrate that monitoring patients via a web application enables the detection of at least 30% more significant events occurring between 2 systematic follow-up consultations with the specialist using an electronic questionnaire.

**Methods:**

We conducted a prospective, randomized phase 3 trial comparing the impact of web-based follow-up (experimental arm) with a standard follow-up (control arm). The trial was based on a 2-step triangular test and was designed to have a power of 90% to detect a 30% improvement in the detection of significant events. A significant event was defined as a relapse, progression, or a serious adverse event. The study covered the follow-up period after completion of first-line treatment or relapse (24 months). Eligible patients were aged 18 years and older and had lymphoma at a high risk of relapse. In the experimental arm, patients received a 16-symptom questionnaire by email every 2 weeks. An email alert was sent to the medical team based on a predefined algorithm. The primary objective was assessed after the inclusion of the 40th patient. The study was continued for the duration of the analysis.

**Results:**

A total of 52 patients were included between July 12, 2017, and April 7, 2020, at 11 centers in France, with 27 in the experimental arm and 25 in the control arm. The median follow-up was 21.3 (range 1.3‐25.6) months, and 121 events were reported during the study period. Most events occurred in the experimental arm (83/119, 69.7%) compared with 30.2% (36/119) in the control arm. A median number of 3.5 (range 1-8) events per patient occurred in the experimental arm, and 1.8 (range 1-6) occurred in the control arm (*P*=.01). Progression and infection were the most frequently reported events. Further, 19 patients relapsed during follow-up: 6 in the experimental arm and 13 in the control arm (*P*<.001), with a median follow-up of 7.7 (range 2.8‐20.6) months and 6.7 (range 1.9‐16.4) months (*P*=.94), respectively. Statistical analysis was conducted after including the 40th patient, which showed no superiority of the experimental arm over the control arm. The study was therefore stopped after the 52nd patient was enrolled.

**Conclusions:**

The primary objective was not reached; however, patient-reported outcome measures remain essential for detecting adverse events in patients with cancer, and the electronic monitoring method needs to demonstrate its effectiveness and comply with international safety guidelines.

## Introduction

Relapse or progression is a major event in the management of lymphoma. Predictive factors for relapse include histological subtype, extranodal involvement, high metabolic volume, and elevated serum lactate dehydrogenase (LDH) levels [1]. Early detection of relapse correlates with survival. In most cases, relapse is detected by the appearance of symptoms, clinical signs, or biological abnormalities [2-4]. Repeated surveillance computed tomography (CT) detects asymptomatic recurrence in only 1.7% of patients and increases the risk of secondary cancers because of radiation overexposure [5-8]. Circulating tumor DNA monitoring may be used to detect early recurrence before the onset of symptoms; however, this method has not been validated [9]. Electronic patient-reported outcome measures (ePROMs) based on the Common Terminology Criteria for Adverse Events have emerged as a method of early detection. This has increased survival rates in some cases (locally advanced lung cancer) [10]. ePROMs affect early event detection and overall survival in patients with cancer [11-15]; however, such evidence is lacking for patients with lymphoma. In this study, we compare the effect of web-based follow-up with that of standard follow-up.

## Methods

### Overview

We conducted an open-label, longitudinal, prospective study between July 12, 2017, and April 7, 2020, at 14 centers in France.

### Ethical Considerations

This study was conducted according to the 1975 Declaration of Helsinki, revised in 2008, and the guidelines of the International Conference on Harmonization of Good Clinical Practice in Biomedical Research. The Ouest II national ethics committee in Angers approved the study on November 8, 2016, and the Agence Nationale de Sécurité du Médicament approved it on November 22, 2016 (approval: 2021–A01670–41). All of the patients provided written informed consent, which included the points of analysis, the method of data collection, and the primary and potential secondary statistical analyses. All patient data were anonymized and no financial compensation was provided.

### Study Population, Inclusion Criteria, and Exclusion Criteria

Patients with lymphoma who were aged 18 years or older and had a high risk of relapse were considered eligible for this trial. They could have T-cell lymphoma in the first partial or complete response, Hodgkin lymphoma in the second partial or complete response, or diffuse large B-cell lymphoma in the first partial or complete response with a revised high International Prognostic Index score (≥3) or in the second partial or complete response. Patients who had undergone autologous stem cell transplantation were not excluded. Eastern Cooperative Oncology Group performance status between 0 and 2, an internet connection, and affiliation to the French social security system were required. Patients were recruited during follow-up consultations by the referring physician at each center.

The exclusion criteria were an initial symptom score <7, progression within 3 months of the last treatment, brain or meningeal involvement, history of another cancer treated within 3 years—with the exception of skin cancer (except melanoma) and in situ cervical cancer—pregnancy, breastfeeding, and any psychiatric pathology that may prevent compliance with the protocol.

An initial symptom score was established in the previous Sentinel study. The e-request algorithm was more sensitive for patients who were not very symptomatic at inclusion and had an initial score of less than 7 (by summing scores from 0 to 3 for symptoms concerning cough, dyspnea, pain, anorexia, and asthenia: 0=no problem, 1=mild problem, 2=moderate problem, and 3=severe problem) [12].

### Randomization

Randomization was planned through minimization once patients were enrolled in the study and programmed using ENNOV Clinical data management software. Patients were randomly assigned 1:1 to a routine follow-up (control arm) or web-mediated follow-up (experimental arm). Stratification was conducted at inclusion according to the center, performance status, autologous stem cell transplantation history, relapse, and lymphoma subtype.

### Follow-Up

Patients were included no later than 3 months at the end of their last treatment. Follow-up was 24 months after enrollment. A medical consultation and a biological assessment were performed every 3 months. In the control arm, CT scans were performed every 6 months. In the experimental arm, scans were performed when medically necessary. Quality of life (QoL) was assessed by 2 questionnaires every 3 months (European Organisation for Research and Treatment of Cancer Quality of Life Questionnaire of Cancer Patients [QLQ-C30] and Patient Health Questionnaire-9 [PHQ-9]) [16,17]. Patient satisfaction with the application was evaluated by an internal questionnaire for patients in the experimental group during the 6-month visit from inclusion.

### Web Application

The Moovcare patient-reported outcome (PRO) system is a class 1 medical device registered by Sivan Innovation, Ltd., with Conformité Européenne marking obtained in July 2017. Versions 1.7 (from July 2017 to October 2019) and 1.8 (from November 2019 to April 2020) were used in this study. “The reimbursed indication of the MOOVCARE Lung device is the early detection of recurrences or complications for patients over the age of 16 with nonprogressive lung cancer after the last medical treatment, regardless of the histological type of the tumor”—an excerpt from the user guide [18]. The indication was validated by the data of the Sentinel Lung study (published in 2019), which demonstrated a survival benefit of 9 months for patients monitored by the application compared to standard monitoring (*P*=.005) [13].

A scientific committee adapted the questionnaire for patients who were being monitored for lymphoma, whereas the technical monitoring and the decision algorithm remained the same.

PRO data were collected using questionnaires sent by the application directly to the patient through a clickable link to their email address. Patients were asked to complete a 16-question self-assessment every 14 days for 24 months after being randomized to the experimental arm (smartphone or email). They were also able to report an event in the web application between the 2 questionnaires. The study coordinators provided them with a short training session on the application. If the patient failed to complete the questionnaire, a reminder was sent after 24 hours, and the health care team contacted the patient as necessary.

The questionnaire included 4 items, namely weight, LDH level (optional), hemoglobin level (optional), and a free comment (for other symptoms or remarks), and 12 following questions:

- Are you tired?
- Have you lost your appetite?
- Are you in pain?
- Are you short of breath?
- Do you feel depressed?
- Do you have a fever (temperature >38.1 °C, checked once at 1-hour intervals)?
- Do you have chills?
- Do you have pimples?
- Are you sweating profusely?
- Are you itching all over your body?
- Have you detected a lump under the skin or a lymph node?
- Have you noticed any abnormal swelling of the face or legs?

Patients were assigned a score based on their symptoms as follows: 0=no problem, 1=mild problem, 2=moderate problem, and 3=severe problem. An alert was triggered in the event of weight loss greater than 2 kg over 1 month, in the event of symptoms rated 3, the presence of fever or night sweats on 2 consecutive occasions, or elevation of serum LDH above 2-fold the normal level, or anemia indicated by hemoglobin levels of <10 g/dL.

**Figure 1.**
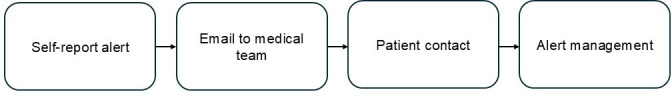
The decision algorithm used in this study.

In the event of an alert triggered by the application, an email was sent to the care team, with a reminder every 24 hours if there was no response (Figure 1). Patients could also report an event by writing a free text.

### Outcome Measures

The primary outcome was to demonstrate that follow-up via a web application could detect more significant events (including relapses) occurring between 2 routine follow-up consultations with the specialist in patients with lymphoma who were at high risk of relapse compared with standard follow-up. Secondary outcomes were overall and progression-free survival at 2 years, relapse rate at 2 years, QoL or patients in both arms, and compliance and satisfaction for the experimental arm.

### Adverse and Significant Events

An adverse event was defined as any symptom reported by the patient either during the protocol consultation in the control arm or, through the application in the experimental arm. An event was considered significant if, the grade was greater (≥2) based on the Common Terminology Criteria for Adverse Events v4.02 or, if it prompted an imaging examination, treatment (of any kind), supportive care, unscheduled consultation, or emergency hospitalization.

#### QoL, Adherence, and Satisfaction

QoL was evaluated using the QLQ-C30 and PHQ-9 questionnaires at inclusion and, follow-up visits at 3, 6, 9, and 12 months. Patient adherence to the use of the web application was assessed according to the number of electronic questionnaires completed. A questionnaire had to be completed every 14 days. Patients completing less than 1 electronic questionnaire every 42 days (6 weeks) were considered noncompliant. Patient satisfaction with web monitoring and the use of the web application was assessed using a self-questionnaire at their 6-month follow-up visit.

#### Data Management and Statistical Analysis

##### Data Management

One electronic case report form (e-CRF; ENNOV Clinical) was created for each patient. The information required by the protocol was collated into the e-CRF, which included the data necessary to confirm compliance with the protocol and detect any major deviations, as well as the data necessary for statistical analysis. The information was collected without mentioning the surname and first name in the e-CRF, with an identification number for the center and a patient number. Only the first letters of the patient’s surname and first were visible. This code was the only patient identifier that appeared in the e-CRF, which made it possible to link e-CRFs to the corrresponding patients.

##### Statistical Analysis

###### Determination of the Size of the Study Population

The trial was based on a 2-step triangular test and was designed to have a power of 90% to detect a 30% improvement in the detection of significant events outside of routine consultations during the 6 months of follow-up with the web application. This is compared with a 60% rate of detection for significant events outside routine consultations among patients randomly assigned to conventional follow-up, with a significance of 5%. This sequential method made it possible to evaluate the application’s effectiveness while controlling the power and type I error (the risk of falsely rejecting our null hypothesis) [19,20]. 40 evaluable patients were to be included per arm, and an interim analysis was to be performed when 20 evaluable patients per arm had 6 months of follow-up. Inclusion was not suspended before the 6-month follow-up.

###### Analysis of Variables, Progression-Free Survival, Overall Survival, and QoL Questionnaires

The analysis of the qualitative variables is presented in terms of numbers and percentages. The analysis of quantitative variables is presented as median or mean (SD) depending on the normality of the variable, whereas the minimum and maximum values are also indicated. The events are described in terms of frequency by etiological type (according to the Medical Dictionary for Egilatory Activities classification) and severity according to the NCI Common Terminology Criteria for Adverse Events version 4.02. For the analysis of censored data (overall survival and other event times), survival curves are plotted based on Kaplan-Meier estimates, and the median survival times and their 95% CIs are presented. For multivariate survival analyses, the Cox semiparametric model was used to calculate the odds ratios, which are presented with 95% CIs. The sensitivity of the application to detect a relapse and significant complications was calculated. The QoL scores were calculated according to the European Organisation for Research and Treatment of Cancer recommendations for the QLQ-C30 [16]. QoL is described for each measurement time, compared at inclusion, and then studied longitudinally using mixed analysis of variance models for repeated measures. PHQ-9 scores were calculated based on the recommendations described at each measurement time and compared at inclusion. Classes proposed in the literature (≤4; 5‐14;>14) were used to describe the patients’ state of depression [17]. Analyses were performed using SAS 9.3 software (SAS Institute, Inc).

## Results

### Study Design

**Figure 2.**
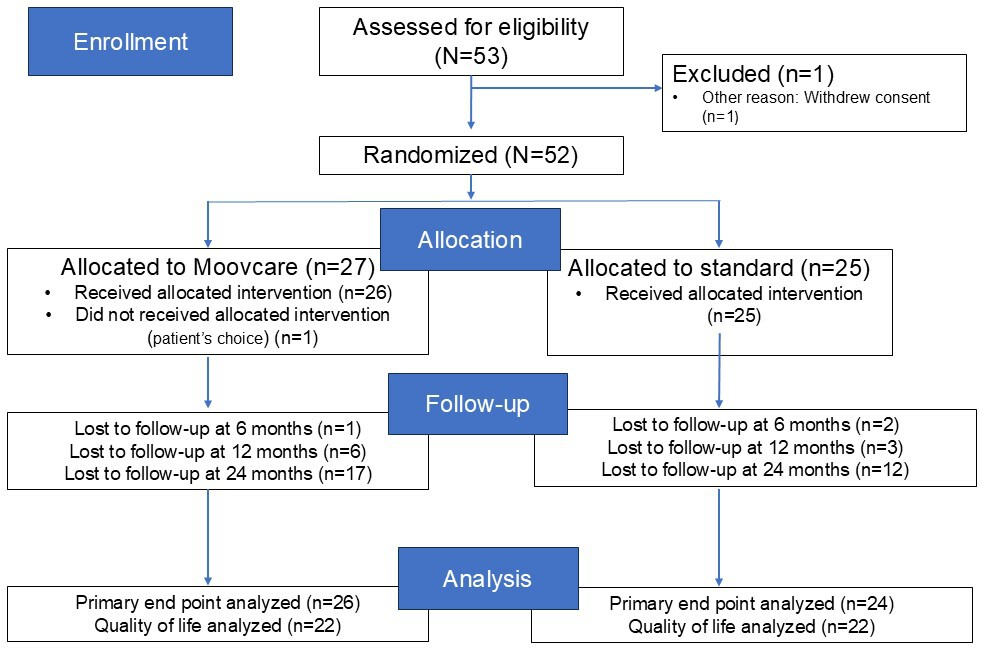
CONSORT (Consolidated Standards of Reporting Trials) flowchart diagram.

A total of 53 patients were included between July 12, 2017, and April 7, 2020, from 14 centers in France: Le Mans (16/52, 31%), Besançon (9/52, 17%), Nantes (6/52, 11%), Bordeaux Bergonié (6/52,11%), Bordeaux Nord (6/52, 11%), Mont de Marson (4/52, 8%), Dijon (1/52, 2%), Grenoble (1/52, 2%), Paris (1/52, 2%), Strasbourg (1/52, 2%), and Vannes (1/52, 2%). One patient withdrew consent before randomization, and 27 patients were randomized to the experimental arm and 25 to the control arm. The median follow-up time was 21.3 (range 1.3‐25.6) months. In total, 26 patients were evaluated at the primary end point in the experimental arm and 24 in the control arm (Figure 2). The Consolidated Standards of Reporting Trials (CONSORT) guideline (Checklist 1) was used to present the results.

### Patient Characteristics

The median age of the entire population was 65.6 (range 20.3‐87.8) years; the median age was 64.3 (range 20.3‐87.8) years in the experimental arm and 69.2 (range 23.9‐84.4) years in the control arm (*P*=.99). Further, 80% of patients (40/50) had large diffuse B-cell lymphoma, 10% (5/50) had T-cell lymphoma, and 10% (5/50) had Hodgkin lymphoma. The 2 arms were well balanced with respect to age and histological subtype (Table 1).

The median time from initial diagnosis to randomization in the study was 7.7 (range 5.0‐170.2) months. The median was 7.5 (range 5.3‐58.2) months for the experimental arm and 7.8 (range 5.0‐170.2) months for the control arm (*P*=.43).

Rituximab, cyclophosphamide, doxorubicin, vincristine, and prednisone were the primary first-line chemotherapeutic regimens. In the experimental arm, 17/26 (65%) patients received a single line of chemotherapy, and 9/26 (35%) received 2 lines. In the control arm, 16/24 (67%) patients received a line of chemotherapy, and 8/24 (33%) received a second line.

**Table 1.** Patient characteristics.

	Total	Web application	Control	*P* value^a^
Sex, n (%)				.80
Male	28 (56)	15 (57.7)	13 (54.2)	
Female	22 (44)	11 (42.3)	11 (45.8)	
ECOG^b^, n (%)				.61
0	19 (38)	9 (34.6)	10 (41.7)	
1	31 (62)	17 (65.4)	14 (58.3)	
Histology, n (%)				.71
Lymphocyte-rich Hodgkin lymphoma	1 (2.0)	1 (3.8)	0 (0)	
Nodular sclerosis and Hodgkin lymphoma	4 (8)	2 (7.7)	2 (8.3)	
ALK-positive anaplastic large T-cell lymphoma	1 (2)	1 (3.8)	0 (0)	
Angio-immunoblastic T-cell lymphoma	2 (4)	1 (3.8)	1 (4.2)	
Peripheral T-cell lymphoma, NOS^c^	1 (2)	0 (0)	1 (4.2)	
Nasal NK^d^ T-cell lymphoma	1 (2)	1 (3.8)	0 (0)	
Centroblastic B-cell lymphoma	5 (10)	4 (15.5)	1 (4.2)	
Diffuse large B-cell, NOS	29 (58)	14 (54)	15 (62.4)	
Primary cutaneous diffuse large B-cell lymphoma, leg-type	1 (2.0)	0 (0)	1 (4)	
Primary mediastinal B-cell lymphoma	1 (2.0)	1 (3.8)	0 (0)	
T-cell-rich large B-cell lymphoma	2 (4.0)	1 (3.8)	1 (4.2)	
Burkitt-like lymphoma	2 (4.0)	0 (0)	2 (8.3)	
Ann-Arbor classification, n (%)				.73
I	2 (4.0)	2 (7.7)	0 (0)	
II	7 (14.0)	3 (11.5)	4 (16.7)	
III	6 (12.0)	3 (11.5)	3 (12.5)	
IV	35 (70.0)	18 (69.3)	17 (70.8)	
Treatment, n				.61
First line				
ABVD^e^	4	2	2	
BEACOPP^f^	1	0	1	
CHOEP^g^	5	3	2	
R-ACVBP^h^	1	0	1	
R-CHOP^i^	27	13	14	
R-DA-EPOCH^j^	1	1	0	
R-MIV^k^	1	1	0	
Radiotherapy	4	4	0	
Other	31	14	17	
Second line				N/A^l^
BEACOPP	1	0	1	
Brentuximab-bendamustine	1	1	0	
Brentuximab-ICE^m^	1	0	1	
DHAP^n^	1	0	1	
MIV^o^	1	0	1	
R-CHOP	1	0	1	
R-DA-EPOCH	1	1	0	
R-DHAP	1	1	0	
R-ESHAP^p^	1	1	0	
Radiotherapy	2	1	1	
Other	14	9	5	

a The *P* value was calculated using chi-sqaure test for qualitative variables, the Wilcoxon test for quantitative variables, and the Fisher test for the lower variables.

b ECOG: Eastern Cooperative Oncology Group.

c NOS: not otherwise specified.

d NK: natural killer.

e ABVD: adriamycin, bleomycin, vinblastine, dacarbazine.

f BEACOPP: bleomycin, etoposide, adriamycin, cyclophosphamide, vincristine, procarbazine, prednisone.

g CHOEP: cyclophosphamide, doxorubicin, vincristine, etoposide, prednisone.

h R-ACVBP: rituximab, doxorubicin, cyclophosphamide, vindesine, bleomycin, prednisone.

i R-CHOP: rituximab, cyclophosphamide, doxorubicin, vincristine, prednisone.

j R-DA-EPOCH: rituximab and dose-adjusted etoposide, prednisone, vincristine, cyclophosphamide, doxorubicin.

k R-MIV: rituximab, mitoxantrone, ifosfamide, etoposide.

l N/A: not assessed (low variables).

m ICE: ifosfamide, carboplatin, etoposide.

n DHAP: dexamethasone, cytarabine, cisplatinum.

o MIV: mitoxantrone, ifosfamide, etoposide.

p R-ESHAP: rituximab, etoposide, cytarabine, cisplatin, methylprednisolone.

### Follow-Up

**Figure 3.**
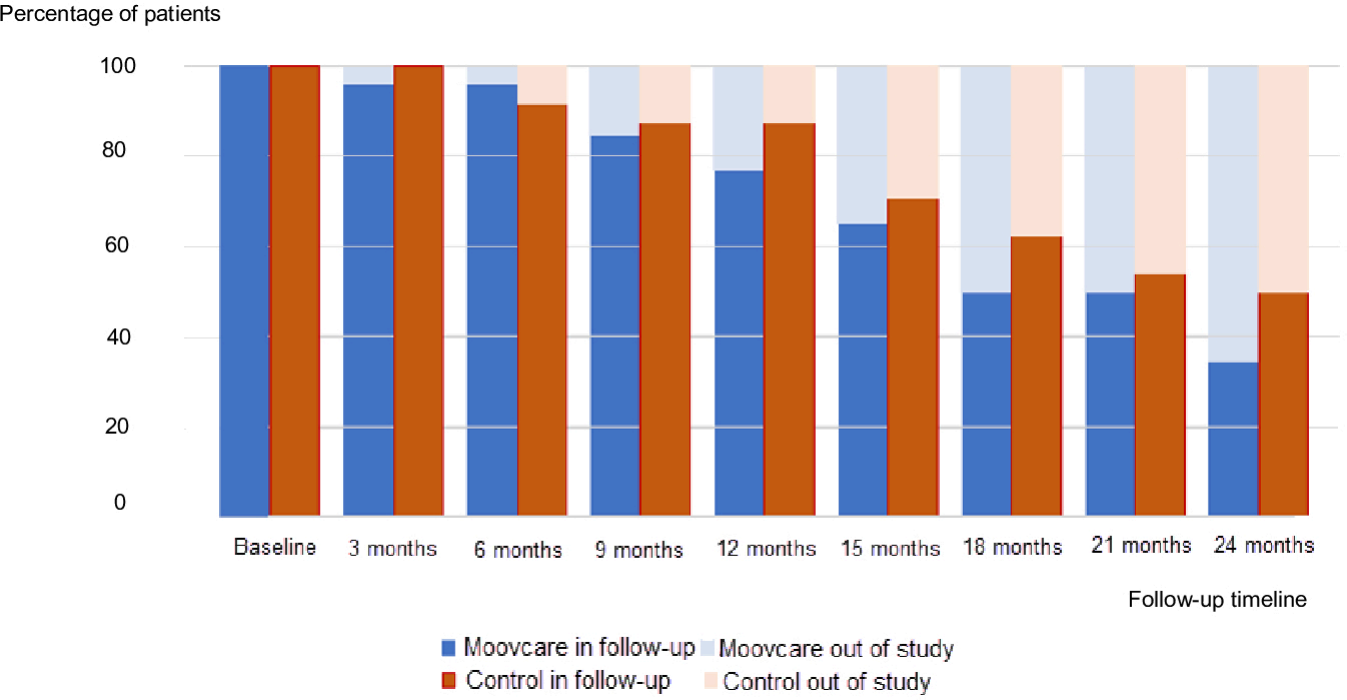
Patient follow-up.

In the experimental arm, 25/26 patients (96.1%) were still included in the study at the 6-month follow-up, 20/26 (76.9%) at 12 months, and 9/26 (34.6%) at 24 months. In the control arm, 22/24 (91.6%) were still being followed at 6 months, 21/24 (87.5%) at 12 months, and 12/24 (50%) at 24 months (Figure 3).

The primary reasons for the loss of follow-up were the planned end of the protocol for 20 patients and the premature termination of the study by the sponsor (19 patients; Table 2).

**Table 2.** Reasons for discontinuing the study.

	Total, n	Web application, n (%)	Control, n (%)
Death	4	3 (75)	1 (25)
Investigator decision	2	0 (0)	2 (100)
Patient decision	1	1 (100)	0 (0)
End of follow-up	20	8 (40)	12 (60)
Missing patient	2	1 (50)	1 (50)
Premature termination	19	11 (58)	8 (42)
Missing data	2	2 (100)	0 (0)

### Events

During the study period, 119 events were reported (Table 3). Most occurred in the experimental arm (83/119, 69.7%) versus 36/119 (30.2%) in the control arm, with a median number of events per patient of 3.5 (range 1-8) in the experimental arm and 1.8 (range 1-6) in the control arm (*P*=.004). In the experimental arm, 47/83 (56.6%) events were reported directly by the medical team after a scheduled consultation, whereas 36/83 (43.3%) were reported through the web application.

In the control arm, 19/36 (52.7%) events were detected during a scheduled consultation, 2/36 (5.6%) during an unscheduled consultation, 9/36 (25%) during a consultation with another specialist, 3/36 (8.3%) during hospitalization, and 3/36 (8.3%) during a patient call.

Progression and infection were the most frequently reported events. 19 patients relapsed during follow-up, with a median follow-up time of 7 (range 1.9‐20.6) months; 6 in the web experimental arm and 13 in the control arm (*P*<.001), with a median follow-up of 7.7 (range 2.8‐20.6) months and 6.7 (range 1.9‐16.4) months (*P*=.94), respectively. 13 patients were treated for relapse; 11 by chemotherapy and 2 by radiotherapy.

30 patients were infected (23 in the experimental arm and 7 in the control arm*; P*=.59): 5 patients had an influenza infection, and the infectious agent was not reported for the others. 14 patients received treatment; 13 with antibiotics, 1 with antivirals, and 1 with antibiotics and antivirals. The grade of adverse events was not available in 60 out of 119 (50.4%) cases, which limited data interpretation.

**Table 3.** Description and classification of events.

	Total, n	Web application, n	Control, n	*P* value^a^
Events	119	83	36	N/A^b^
Number of events per patient	—^c^	3.5	1.8	.004
Grade (progression excluded)	98	75	23	
Grade 1/2	27	19	8	
Grade 3/4	10	7	3	
Unknown	61	49	12	
Progression	21	8	13	—
Suspected by the imaging data	2	2	0	—
Confirmed by biopsy	19	6	13	<.001
Infection	30	23	7	.60
Severity				
Unknown grade	20	15	5	
Grade 1‐2	6	6	0	
Grade 3‐4	4	2	2	
Subtype				
Pharyngitis	4	2	2	
Pneumopathy	4	2	2	
Bronchitis	2	1	1	
Influenzae infection	5	5	0	
Anal collection	1	0	1	
Urinary infection	2	2	0	
Gastroenteritis	3	3	0	
Not specified	9	8	1	
Pain	22	18	4	.13
Abdominal	7	7	0	
Thoracic	3	3	0	
Bone and muscle	5	3	2	
Not specified	7	5	2	
Secondary neoplasia	3	1	2	.41
Melanoma	1	1	0	
Colonic adenocarcinoma	1	0	1	
Uterine neoplasm	1	0	1	
Neurological events	5	4	1	.83
Peripheral neuropathy	2	2	0	
Dizziness	3	2	1	
Thrombosis	3	3	0	N/A^d^
Arterial	1	1	0	
Venous	2	2	0	
Bleeding events	1	1	0	N/A^d^
Epistaxis	1	1	0	
Skin Rash	5	5	0	N/A^d^
Kidney-related events	3	2	1	.99
Increase in creatinine levels	2	1	1	
Kidney lithiasis	1	1	0	
Biological events	3	1	2	.41
Iron deficiency	1	0	1	
Hypercalcemia	1	0	1	
Elevated LDH^e^ levels	1	1	0	
Other	23	17	6	.85
Fatigue	4	3	1	
Dyspnea or cough	3	3	0	
Itching	2	2	0	
Edema	4	4	0	
Gynecomastia	1	1	0	
Jugal cyst	1	0	1	
Colonic polyposis	2	0	2	
Hypertension	1	1	0	
Not specified radiological abnormalities	5	3	2	

a The *P* value was calculated using the chi-square test for qualitative variables, the Wilcoxon test for quantitative variables, and the Fisher test for the lower variables.

b N/A: not assessed (noncomparable values).

c Not applicable.

d N/A: not assessed (low values).

e LDH: lactate dehydrogenase.

### Event Management

#### Overview

Events led to 19 additional medical consultations with the referring hematologist in the experimental arm (alert management resulted in 8 additional medical consultations and 11 without an alert) versus 15 in the control arm (*P*=.99; Table 4). 30 consultations were conducted with other specialists (15 in the experimental arm and 15 in the control arm). For some events, several specialists or referring hematologists were required to manage the patient.

**Table 4.** Management of events.

	Total, n	Web application, n	Control, n	*P*-value^a^
Consultation with the oncologist	31	16	15	N/A^b^
Referral to another specialist	30	15	15	N/A^b^
Imaging (scan)	33	22	11	.91
Hospitalization	8	5	3	.78
Progression	4	1	3	
Thoracic pain	1	1	0	
Myocardial infarction	1	1	0	
Balance disorders	1	1	0	
Respiratory distress	1	1	0	
Medical treatment	39	28	11	.94

a The *P* value was calculated using the chi-square test for qualitative variables, the Wilcoxon test for quantitative variables, and the Fisher test for the lower variables.

b N/A: not assessed.

Emergency hospitalization was required for 8 patients (4 for progression, 1 for myocardial infarction, 1 for respiratory distress, 1 for thoracic pain, and 1 for balance disorder), with no difference between the 2 arms (*P*=.78). 22 scans were performed in the web experimental arm versus 72 in the control arm (61 scanners scheduled for follow-up and 11 not scheduled for events*; P*<.001).

39 events required treatment, with a total of 24 patients receiving medical treatment (16 in the experimental arm and 8 in the control arm; *P*=.11; Table 5). 56 prescriptions were filled (37 in the web experimental arm and 19 in the control arm*; P*=.64).

**Table 5.** Pharmacological treatment of events.

	Total, n	Web application, n	Control, n	*P* value^a^
Total	56	37	19	.64
Antiinfection drugs	20	10	10	N/A^b^
Analgesics	5	3	2	N/A^b^
Neurological treatment	2	2	0	N/A^b^
Gastroenterological treatment	7	7	0	N/A^b^
Cardiological treatment	6	4	2	N/A^b^
Anticoagulant treatment	2	1	1	N/A^b^
Systemic corticoids	1	0	1	N/A^b^
Other not specified	13	10	3	N/A^b^

a The *P* value was calculated using the chi-square test for qualitative variables, the Wilcoxon test for quantitative variables, and the Fisher test for the lower variables.

b N/A: not assessed.

#### QoL and Depression

The patients were asked to complete the QLCQ-30 questionnaire every 3 months for 1 year. 44 patients completed at least 2 QoL questionnaires during the study; 22 patients per arm (ie, 22/26, 84.6% in the experimental arm and 22/24, 91.6% in the control arm). The higher the score, the poorer the QoL (maximum score: 114). The median scores did not differ between the 2 groups at 12 months with it being; 45 in the experimental arm (range 39-61) and 44 in the control arm (range 30-69*; P*=.94). Regarding depression, 42 patients completed the questionnaire (21 per arm; ie, 21/26, 80.7% in the experimental arm and 21/24, 87.5% in the control arm]. The score was 1.0 (range 0‐15) in the experimental arm versus 1.5 (range 0‐13) in the control arm (*P*=.73).

#### Satisfaction (Experimental Arm)

In total, 20 patients in the experimental arm completed the satisfaction questionnaire (20/25, 80%). Further, 95% (19/20) of patients who responded to the satisfaction questionnaire were satisfied and reassured by the application, whereas 90% (18/20) felt better informed.

#### Survival

**Figure 4.**
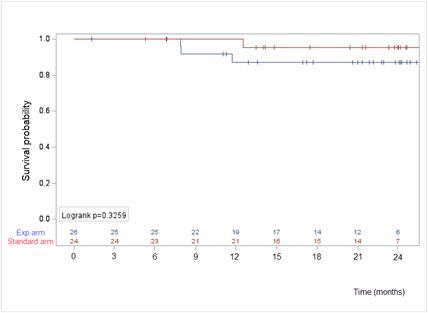
Overall survival. Exp.: experimental.

**Figure 5.**
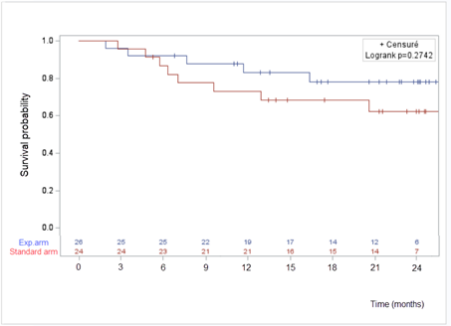
Progression-free survival. Exp.: experimental.

Four patients died during the trial; 3 in the experimental arm and 1 in the control arm (*P*=.34). Overall survival at 12 months was 87.1% in the experimental arm (95% CI 65%-95.7%) and 95.2% in the control arm (95% CI 70.7-99.3%; *P*=.32; Figure 4). Progression-free survival at 12 months was 83.2% in the experimental arm (95% CI 61%-93.3%) and 68.5% in the control arm (95% CI 44.9%-83.6%; *P*=.27; Figure 5).

#### Protocol Deviations and Technical Failures in the Experimental Arm

Three alerts were not handled by the care team within the required time frame and were subsequently classified as minor (grade 1).

The automatic sending of questionnaires was stopped for 9/26 (35%) patients. For 3 patients, the questionnaires were sent in paper form. For the other 6, no solution could be found despite repeated interventions by the electronic application’s technical department. One of these patients suffered 2 major events: myocardial infarction and relapse. These 2 events were not reported in the electronic application. Because of these technical problems, compliance could not be assessed.

#### Outcomes

An interim analysis was performed based on the protocol when the first 40 patients reached 6 months of follow-up. The results of this analysis did not reject the null hypothesis, which stated that there was no difference in the diagnosis of events between the 2 arms. The Sentinel Lymphoma Study Committee met on March 11, 2021, to oversee the analysis of the primary outcome, which indicated no difference in the diagnosis of significant events. A decision was made at the end of the meeting to discontinue the trial early. Based on the protocol, the study was terminated on March 15, 2021, following the sponsor’s decision.

## Discussion

### Principal Findings

In this study, there is no difference in the occurrence of significant events between the 2 arms (median number per patient of 3.5 in the experimental arm and 1.8 in the control arm; *P*=.004). Progression, infection, and pain were the most frequently reported events. Patient satisfaction was very high and the patients felt reassured to have electronic monitoring. The patients included in the experimental arm underwent fewer scans compared with those in the control arm, without impacting overall survival, despite a short follow-up (*P*<.001).

### Strengths and Limitations

First, the primary outcome of a 30% superiority of reporting significant events in the experimental arm has been overly optimistic. Thus, reducing the end point would have led to a substantial increase in the number of included patients. The number of events was probably not the best criterion for evaluating the effectiveness of remote monitoring. An improvement in QoL or a reduction in the risk of relapse would likely have been more relevant [13,15].

Second, technical problems with the web application occurred (electronic questionnaires not received, with major biases in event reporting). The incidents were not expected because of the experience of the software developer (Moovcare, Sivan Innovation, Ltd); however, there was a change in the technical team between this publication on lung cancer and the start of our study [11-13]. The blocking of automatic questionnaires required 42 direct interventions by clinical study investigators with calls to the patient (firewalls and spam). IT support did not correct these recurring anomalies, despite the changes to the application in November 2019 (5 patients were included in the experimental arm after this date). These operational problems resulted in 15 meetings without resolution of the problems, with an average response time of 4.6 months from technical support (frequent changes to contact persons). As a result, the events in the experimental arm were not reported correctly, leading to study bias. The final report has been sent to the Agence Nationale de Sécurité du Médicament on June 29, 2021.

Finally, only 43% of the events were declared by the application in the experimental arm, which would indicate a problem with patient training.

### Comparison With Prior Work

PROs are underestimated in clinical practice and trials for patients with lymphoma and have most often consisted of paper-based QoL questionnaires [21]. The measurement of PROs via electronic questionnaires has subsequently been evaluated in randomized trials with a low representation of patients with lymphoid malignancies [22]. However, Maguire et al [23] demonstrated that real-time electronic monitoring of symptoms was feasible during an initial chemotherapy cycle in patients with solid tumors and lymphoma, with a reduction in the intensity of side effects and anxiety, compared with a control group.

### Future Directions

Proposals for the future include improving the study design by limiting the patient population to a single type of lymphoma, defining the objective to demonstrate an improvement in morbidity and possibly reduce cost, and guaranteeing the reliability of the electronic application. PROMs require standardization of analysis for comparative purposes. Therefore, it is necessary to regulate the use of health care applications to avoid malfunction and abuse [24]. Denis and Krakowski [25] defined 20 criteria of effectiveness, safety, and functionality that should govern the development of ePROMs. Telemonitoring applications should strive to improve patient compliance and prevent patients from dropping out due to a lack of understanding or receiving excessive notifications [26]. Telemonitoring applications must evolve with therapeutic innovations and be regularly reevaluated to demonstrate their long-term benefits on a larger scale [27]. Finally, guidelines are recommended for the design of clinical trials to evaluate the effectiveness of electronic solutions [28-30].

### Conclusions

Sentinel Lymphoma is the first randomized phase 3 trial to evaluate the effect of remote monitoring on the detection of significant events in patients with hematological malignancies. Progression, infection, and pain were the most frequently reported events. Despite a high number of events (83 in the experimental arm against 36 in the control arm), the difference was not significant. A more targeted population, a more precise objective, and better security for remote surveillance solutions are recommended for subsequent projects.

## Supplementary material

10.2196/65960 Checklist 1 CONSORT eHEALTH checklist (V 1.6.1).

## Abbreviations

CONSORT: Consolidated Standards of Reporting Trials
CT: computed tomography
e-CRF: electronic case report form
ePROM: electronic patient-reported outcome measure
LDH: lactate dehydrogenase
PHQ-9: Patient Health Questionnaire-9
PRO: patient-reported outcome
PROM: patient-reported outcome measure
QLQ-C30: Quality of Life Questionnaire of Cancer Patients
QoL: quality of life
